# Genetic diversity and population structure of wheat in India and Turkey

**DOI:** 10.1093/aobpla/plv083

**Published:** 2015-07-17

**Authors:** Mohd Kamran Khan, Anamika Pandey, George Thomas, Mahinur S. Akkaya, Seyit Ali Kayis, Yusuf Ozsensoy, Mehmet Hamurcu, Sait Gezgin, Ali Topal, Erdogan E. Hakki

**Affiliations:** 1 Department of Soil Science and Plant Nutrition, University of Selcuk, Konya 42079, Turkey; 2 Department of Molecular and Cellular Engineering, Sam Higginbottom Institute of Agriculture, Technology and Sciences, Allahabad, India; 3 Department of Chemistry, Middle East Technical University, Ankara 06800, Turkey; 4 Department of Biostatistics, Karabuk University, Karabuk 78050, Turkey; 5 Department of Biometrics and Genetics, Cumhuriyet University, Sivas, Turkey; 6 Department of Field Crops, University of Selcuk, Konya 42079, Turkey

**Keywords:** Genetic diversity, molecular markers, ploidy level, population structure, wheat

## Abstract

Genetic diversity among plant species offers prospects for improving the plant characteristics. Its assessment is necessary to help tackle the threats of environmental fluctuations and for the effective exploitation of genetic resources in breeding programmes. Although wheat is one of the most thoroughly studied crops in terms of genetic polymorphism studies, phylogenetic affinities of Indian and Turkish *Triticum* species have not been assessed to date. In this study, genetic association of 95 tetraploid and hexaploid wheat genotypes originating from India and Turkey was determined for the first time. Combined analysis of random amplified polymorphic DNA and inter-simple sequence repeat markers disclosed 177 polymorphic bands, and both the dendrogram and two-dimensional scatterplot showed similar groupings of the wheat genotypes. Turkish hexaploid varieties were basically divided into two clusters, one group showed its close association with Indian hexaploid varieties and the other with Indian tetraploid varieties. Analysis of molecular variance revealed high (77 %) genetic variation within Indian and Turkish populations. Population structure analysis elucidated distinct clustering of wheat genotypes on the basis of both geographical origin and ploidy. The results revealed in this study will support worldwide wheat breeding programmes and assist in achieving the target of sustainable wheat production.

## Abstract (precis)

Genetic diversity assessment plays an important role in plant improvement. It becomes more significant when evaluation is done at different ploidy and geographical origin levels. The present study provides a better understanding of the genetic association of Indian and Turkish hexaploid and tetraploid wheat. The Turkish hexaploid population demonstrated its close association with Indian hexaploid and tetraploid varieties. This confirmed their relatedness within the diverse gene pool. The results revealed in this study can be effectively used by breeders and evolutionary biologists for the development of genetically diverse, promising and healthier wheat varieties.

## Introduction

Genetic variability in natural plant populations holds the potential to deal with multiple biotic and abiotic stresses. The potential to select a superior line increases with genetic diversity, the discovery of which becomes an important tool in plant breeding. On depletion of genetic variability, plants are unable to cope with unfavourable environmental conditions or pathogens and pests. Diversity studies also facilitate the conservation and management aims of a particular plant species. For the effective use of genetic diversity in plant breeding, knowledge of its extent and distribution plays a crucial role. Considering its significance, a large number of studies have been performed to estimate genetic diversity employing various methodologies in multiple plant species. Assessment of genetic variability employing molecular markers has proved to be a keystone to understanding the genomic constitution, categorizing the genes responsible for important traits, the classification and conservation of genetic variation in plant germplasm and developing selective proliferation approaches for plant propagation.

Wheat production in developing countries moved from defective to surplus (Pingali 2012) during the Green Revolution (Gollin *et al*. 2005). Being a good source of carbohydrate, protein, sugar, fat, fibre and minerals, it provides half of the energy requirements of the human population (Simmonds 1989; Shewry 2007; Topping 2007). A constantly rising population demands an increment in wheat production (Ehrlich 1975; Evans 1998; Tilman *et al*. 2011; Ray *et al*. 2013). As the crop already covers a wide agricultural area, there is a negligible possibility of area expansion (Young 1999; Bruinsma 2003; Cassman *et al*. 2003). Wheat faces multiple demands including its growth under warmer conditions (Vermeulen *et al*. 2012; Wheeler 2012), fighting various diseases (Summers and Brown 2013), reduced energy input for sustainable growth (Ziaei *et al*. 2015) and high nutritional quality (Shewry 2007, 2009). Considering this situation, Lynch (2007) has suggested a need for a ‘second green revolution’. This second green revolution must place emphasis on the utilization of inherent resources and the thorough understanding of genetic diversity.

During the course of evolution, wheat gained sufficient genetic diversity along the road from einkorn to bread wheat. Today, however, its diversity is weakening due to repeated cultivation of landraces for specific characters, narrow adaptation, farmers' varietal selection and the requirement of uniform varieties in industrial seed grain processing (Bellon 1996; Smale 1997; Heal *et al*. 2004). Implementation of high-yielding commercial varieties played an important part in loss of genetic variation. This depletion has now encouraged the use of genetic resources in wheat breeding programmes.

Genetic diversity is crucial for adaptability and survival of wheat species against the threat of disease attack/onset (Fu and Somers 2009). If all the individuals of a population are identical, they will behave similarly to a stress condition and potentially be equally unable to cope with the situation. Hence, it is beneficial to assess the genetic diversity at a particular level that may facilitate the efficient exploitation of the germplasm. Furthermore, in addition to the fact that genetic diversity plays a part in the development of high-yielding bread wheat varieties, issues like the spread of coeliac disease necessitate the development of new genetic variants of tetraploid wheat (van Herpen *et al*. 2006; van den Broeck *et al*. 2010).

Polyploidy and genome evolution of wheat are also partially responsible for maintaining its genetic diversity. In a review, Wendel (2000) shed light on several aspects of the genome duplication and divergence leading to the development of evolutionary genetic diversity. Polyploidy resulting from hybridization leads to gene duplication across the entire genome and thus underlies the emergence of genetic variation. The agriculturally important phenomenon of hybrid vigour in polyploids is a consequence of genetic variability. As wheat is a polyploid species, it is beneficial to include tetraploid and hexaploid varieties in genetic variability assessment programmes. Such assessment programmes are imperative for managing populations by identifying the breeding genotypes. For a long time, depiction of diversity was dependent on morphological characterization (Tesfaye *et al*. 1991; Marić *et al*. 2004; Takumi *et al*. 2009). But due to the influence of environmental conditions and changes during developmental stages, morphological traits are considered unreliable for diversity estimation, mainly for closely associated populations.

Momentous progress in molecular genetics benefitted our understanding of the wheat genome and provided approaches for breeding. With the expansion of novel technologies like molecular markers, researchers utilized a range of *Triticeae* species for genotypic identification (Khan *et al*. 2014). Molecular marker techniques vary from each other in data generation efficiency and the genome area covered in the study. Selection of the type of marker tool for a study depends on the target crop and the issue. For example, random amplified polymorphic DNA (RAPD) markers are known for their simplicity, cost efficiency, fast polymorphism assessment, no prior information of DNA sequences being required and extensive coverage of the intact genome being possible. However, due to low reproducibility of the RAPD system, expense of the amplified fragment length polymorphism (AFLP) marker system and requirement of prior information about DNA sequences in SSR analysis, another dominant marker system, inter-simple sequence repeats (ISSRs), was included in the study. Due to high annealing temperature and extended sequence in comparison to RAPD markers, ISSR primers can produce more reproducible and reliable band patterns. Inter-simple sequence repeat markers are employed for distinguishing DNA on the basis of single base variation or insertions and deletions, and are equivalent to the SSR system in reproducibility. These markers are widely implemented for DNA fingerprinting, identification of species association, genetic variability studies and for recognizing the geographic origin of different plant species along with their ploidy status (Vierling and Nguyen 1992; Joshi and Nguyen 1993*a*, *b*; Autrique *et al*. 1996; Nagaoka and Ogihara 1997; Sun *et al*. 1998; Fahima *et al*. 1999; Pasqualone *et al*. 2000; Pecetti *et al*. 2001; Bered *et al*. 2002; Mukhtar *et al*. 2002; Pujar *et al*. 2002; Mandoulakani *et al*. 2003; Marić *et al*. 2004; Bhutta *et al*. 2005; Motawei *et al*. 2007; Carvalho *et al*. 2009; Najaphy *et al*. 2011; Saleh 2012; Izzatullayeva *et al*. 2014). Hence, in the present study, RAPD and ISSR were chosen among the various marker systems to yield the benefits of both the techniques, diminishing their drawbacks and increasing the credibility of our results.

India and Turkey play crucial roles in supporting food security through wheat production. India holds first and second place in wheat growing area and production, respectively. It has become a priority to replace the uniform high-yielding varieties spread during the Green Revolution with diverse high-quality varieties. Turkey is found to be the place of origin of both tetraploid and hexaploid wheat domestication (Heun *et al*. 1997; Nesbitt and Samuel 1998; Dubcovsky and Dvorak 2007) and India is known to be the centre of origin of some promising varieties. An assessment of genetic variability and association of tetraploid and hexaploid wheat varieties from the two developing countries would be of immense benefit to wheat improvement programmes.

Association and contrast among the wheat cultivars from different countries can provide a useful overview on the evolutionary record of the genotypes and, hence, can facilitate the reach of breeding improvement. Although a number of genetic similarity studies were conducted on diverse wheat germplasm using ISSR and RAPD marker systems, phylogenetic association of Indian and Turkish *Triticum* species has not been documented to date. The present study represents the first effort in this direction, its objectives being to gain a better understanding of the genetic association and population structure of Indian and Turkish wheat on the basis of both geographical origin and ploidy. The share of the genetic variations within and among populations was also revealed so that the information provided can be effectively used by scientists for the development of genetically diverse, promising and healthier wheat varieties.

## Methods

### Study materials

The object of the present diversity study was a collection of 95 Indian and Turkish wheat genotypes including tetraploid (*Triticum turgidum* ssp. *durum*) and hexaploid (*Triticum aestivum* L.) wheat cultivars (Table 1). Well-known varieties were chosen for the experiment to facilitate the use of results in future breeding programmes.

**Table 1.** Name and ploidy of 95 Indian and Turkish wheat genotypes used in the study.

Sl. no.	Name of genotype	Genotype number	Ploidy	Origin
1	30_KR-8	G1	6X	India
2	AAI_2	G2	6X	India
3	AKAW_4006	G3	6X	India
4	AKDW_2997	G4	4X	India
5	DBW_14	G5	6X	India
6	DBW_39	G6	6X	India
7	DDK_1025	G7	6X	India
8	DT_132	G8	4X	India
9	GW_03-12	G9	6X	India
10	GW_03-2	G10	6X	India
11	GW_03-3	G11	6X	India
12	GW_03-4	G12	6X	India
13	GW_03-9	G13	6X	India
14	HD_2177	G14	6X	India
15	HD_2236	G15	6X	India
16	HD_2270	G16	6X	India
17	HD_2307	G17	6X	India
18	HD_2329	G18	6X	India
19	HD_2380	G19	6X	India
20	HD_2402	G20	6X	India
21	HD_2501	G21	6X	India
22	HD_2643	G22	6X	India
23	HD_2881	G23	6X	India
24	HUW_12	G24	6X	India
25	HUW_251	G25	6X	India
26	HUW_37	G26	6X	India
27	HUW_468	G27	6X	India
28	HUW_533	G28	6X	India
29	HUW_55	G29	6X	India
30	K_01006	G30	6X	India
31	K_0204	G31	6X	India
32	K_616	G32	6X	India
33	K_8020	G33	6X	India
34	K_86	G34	6X	India
35	K_88	G35	6X	India
36	K_911	G36	6X	India
37	KALYANSONA	G37	6X	India
38	KBD_65	G38	4X	India
39	KBD_821	G39	4X	India
40	KBD_921	G40	4X	India
41	KBD_922	G41	4X	India
42	KBD_925	G42	4X	India
43	KBD_9452	G43	4X	India
44	KBD_9915	G44	4X	India
45	KD_9851	G45	4X	India
46	KLP_306	G46	6X	India
47	KLP_307	G47	6X	India
48	KLPD_1106	G48	4X	India
49	NAW_1448	G49	6X	India
50	NIDW_295	G50	4X	India
51	NW_1076	G51	6X	India
52	NW_2036	G52	6X	India
53	PBW_550	G53	6X	India
54	RAJ_1482	G54	6X	India
55	RAJ_1555	G55	4X	India
56	RAJ_3072	G56	6X	India
57	RAJ_3077	G57	6X	India
58	RAJ_3777	G58	6X	India
59	RAJ_4027	G59	6X	India
60	RAJ_4037	G60	6X	India
61	RAJ_4120	G61	6X	India
62	RAJ_6560	G62	4X	India
63	RD_1008	G63	4X	India
64	RD_1063	G64	4X	India
65	RD_1093	G65	4X	India
66	RD_1097	G66	4X	India
67	SAW_327	G67	6X	India
68	SAW_337	G68	6X	India
69	SAW_94	G69	6X	India
70	SONALIKA	G70	6X	India
71	UP_2338	G71	6X	India
72	UP_2511	G72	6X	India
73	UP_2525	G73	6X	India
74	UP_2696	G74	6X	India
75	VEERI	G75	6X	India
76	VL_832	G76	6X	India
77	WR_1381	G77	6X	India
78	WR_1408	G78	6X	India
79	WR_1421	G79	6X	India
80	BAYRAKTAR 2000	G80	6X	Turkey
81	SEVAL	G81	6X	Turkey
82	KENANBEY	G82	6X	Turkey
83	BEZOSTAJA 1	G83	6X	Turkey
84	GÜN_91	G84	6X	Turkey
85	KONYA_2002	G85	6X	Turkey
86	AKBUĞDAY	G86	6X	Turkey
87	GEREK_79	G87	6X	Turkey
88	KIRAÇ_66	G88	6X	Turkey
89	ESER	G89	6X	Turkey
90	SÖNMEZ 2001	G90	6X	Turkey
91	HARMANKAYA 99	G91	6X	Turkey
92	KINACI 97	G92	6X	Turkey
93	YÜREĞIR 89	G93	6X	Turkey
94	ALTAY 2000	G94	6X	Turkey
95	LÜTFIBEY	G95	6X	Turkey

### Plant genomic DNA extraction

Two to three weeks grown seedlings were utilized for total wheat DNA extraction following the cetyltrimethylammonium bromide (CTAB) method (Doyle 1990) with some modifications. Initially, cells were disrupted and purified with 2 % CTAB buffer and 10 μL RNase A, respectively, followed by incubation at 65 °C. This was followed by protein extraction employing phenol : chloroform : isoamyl alcohol and finally the CTAB–DNA complex was precipitated with isopropanol. The DNA pellet was twice washed with 70 % ethanol, dried and ultimately, dissolved in 100 μL DNase–RNase-free water. Purified DNA quantity and quality were verified using spectrophotometry and 1 % agarose gel electrophoresis, respectively. The DNA samples were diluted to a concentration of 50 ng μL^−1^ as templates for polymerase chain reactions (PCRs).

### Inter-simple sequence repeats analysis

Twenty-seven ISSR primers (Metabion) were examined for distinguishing the polymorphism patterns, and among those 10 primers showed positive outcomes (Table 2) against chosen wheat varieties. Every PCR mixture of 25 μL contained 2.5 μL of 10× *Taq* buffer containing ammonium sulfate (except ISSR F3 where KCl was used), 3 μL of 25 mM MgCl_2_, 0.4 μL of 25 mM dNTP, 0.5 μL of 10 μM primer, 1.5 units of *Taq* DNA Polymerase and 100 ng of template DNA. The two-step ISSR–PCR reactions were performed in a Eppendorf Master Cycler. The physical reaction conditions and the number of initial and final PCR cycles were optimized for each individual ISSR primer.

**Table 2.** Characteristics and polymorphism revealed by ISSR primers for 95 wheat genotypes used in the study.

ISSR primer	Sequence	Melting temperature (*T*_m_)	Total number of bands	Polymorphic bands	Per cent polymorphism detected
ISSR F3	5′-(AG)_8_ CG-3′	56.0	8	7	87.5
ISSR F4	5′-(AG)_8_ TG-3′	53.7	12	12	100
ISSR F9	5′-(GAA)_5_-3′	39.6	13	12	92.3
ISSR M1	5′-(AGC)_6_ G-3′	63.1	12	11	91.6
ISSR M2	5′-(ACC)_6_ G-3′	63.1	14	14	100
ISSR M3	5′-(AGC)_6_ C-3′	63.1	17	16	94.1
ISSR M8	5′-(AC)_9_ G-3′	56.7	13	12	92.3
ISSR M9	5′-(AC)_8_ CG-3′	56.0	13	13	100
ISSR M12	5′-(GACAC)_4_-3′	61.4	6	6	100
ISSR M17	5′-CAG (CA)_8-_3′	56.7	8	7	87.5
Total			116	110	94.8

### Random amplified polymorphic DNA analysis

For RAPD reactions, a total of 43 primers (MWG Biotech-AC) were screened for polymorphism using selected genotypes, and 10 primers were selected for the final reactions (Table 3). The PCR mixture contained 1.5 μL of 10× *Taq* buffer with ammonium sulfate, 2.5 μL of 25 mM MgCl_2_, 3 μL of 1 mM dNTP, 3 units *Taq* DNA polymerase (Thermoscientific), 1.5 μL of 5 μM RAPD primer and 50 ng of template DNA in a total volume of 15 μL. Polymerase chain reaction amplifications were carried out utilizing a Eppendorf Master Cycler with initial denaturation at 94 °C for 3 min, followed by repeated cycles of denaturation at 94 °C for 45 s, annealing as per the primer's melting temperature for 1 min and primer extension at 72 °C for 1 min. On completion of the repeated number of cycles, final extension was performed at 72 °C for 10 min.

**Table 3.** Characteristics and polymorphism revealed by RAPD primers for 95 wheat genotypes used in the study.

RAPD primer	Sequence	Melting temperature (*T*_m_)	Total number of bands	Polymorphic bands	Per cent polymorphism detected
cRAPD1	5′-GAA ACG GGT G-3′	32	6	4	66.6
cRAPD2	5′-GTG ACG TAG G-3′	32	12	11	91.6
RAPD B3	5′-GTG ACG TAG G-3′	34	9	7	77.7
RAPD B4	5′-CTC ACC GTC C-3′	34	6	5	83.3
RAPD B5	5′-GAC GGA TCA G-3′	32	11	10	90.9
RAPD B10	5′-CTA CTG CGC T-3′	32	7	6	85.7
RAPD B13	5′-TTC AGG GTG G-3	32	5	3	60.0
RAPD L2	5′-GTT TCG CTC C-3′	32	8	7	87.5
RAPD L4	5′- AAG AGC CCG T-3′	32	11	9	81.8
RAPD L6	5′-CCC GTC AGC A-3′	34	7	5	71.4
Total			82	67	81.7

### Data analysis

All the ISSR- and RAPD-based PCRs were repeated three times for the identification of reproducible amplified bands. Amplified fragments were counted from smaller to larger size. A binary data matrix was obtained by scoring the gel as 1 and 0 to show the presence and absence of bands, respectively. Information capacity of the primers and polymorphism content of the genotypes were estimated by calculating the total number of bands and of polymorphic bands. The binary data matrix was used to obtain the similarity matrix depending on simple matching (SM) coefficient by Numerical Taxonomy and Multivariate Analysis System (NTSYS-PC) version 2.02e software (Rohlf 1997). This similarity matrix was utilized in R software for constructing a combined dendrogram of RAPD and ISSR.

On the basis of SM coefficients, the similarity matrix was double centred using the DCENTER module of NTSYS-PC. Then eigen analyses were performed using the EIGEN module of NTSYS-PC to construct two-dimensional scatterplots by the R package. Scatterplots were drawn for the substantiation of the dendrograms and verification of genotypes clustering according to both ploidy and geographical origin.

To explain the population structure of Indian and Turkish wheat genotypes, analysis of molecular variance (AMOVA) was performed using GenAlEx 6.5 software (Peakall and Smouse 2006, 2012) with 1000 permutations. The programme was used for the determination of variance components and estimating the total variation within and among the populations.

Bayesian model-based clustering with assumed *K* populations was employed for genetically homogenous group estimation in Indian and Turkish wheat germplasm. A parameter of 50 000 burn-in period and 100 000 Markov Chain Monte Carlo replication, along with the admixture model and correlated allele frequencies, was used in STRUCTURE, version 2.3.4 (Pritchard *et al*. 2000; Falush *et al*. 2003, 2007; Hubisz *et al*. 2009). A total of 10 independent runs were performed for each value of *K* (from 1 to 4 assumed) (Evanno *et al*. 2005). For the determination of the best possible *K* value elucidating the genetically distinctive clusters in the data, the Structure Harvester v6.0 (Earl and vonHoldt 2012) programme was used implementing parameters described by Evanno *et al*. (2005).

## Results

### Genetic diversity

**Figure 1.**
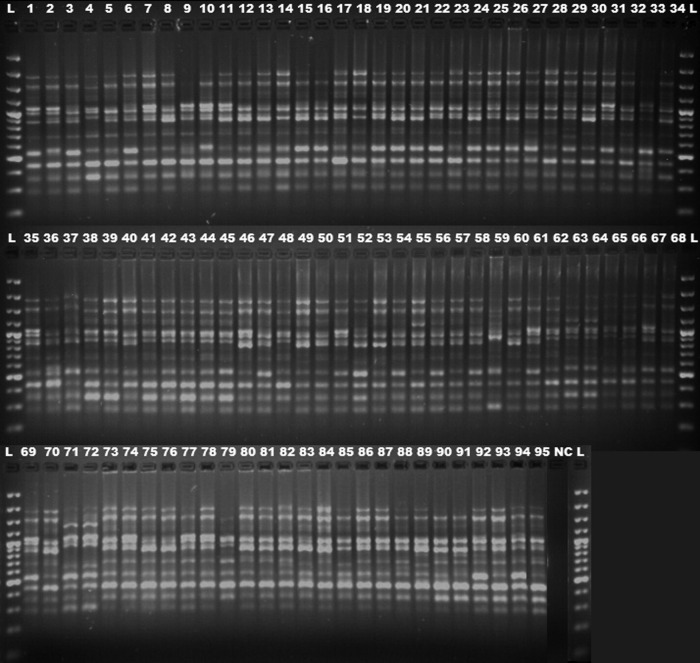
The inter-simple sequence repeat M3 primer amplification profile of 95 Indian and Turkish wheat genotypes.

**Figure 2.**
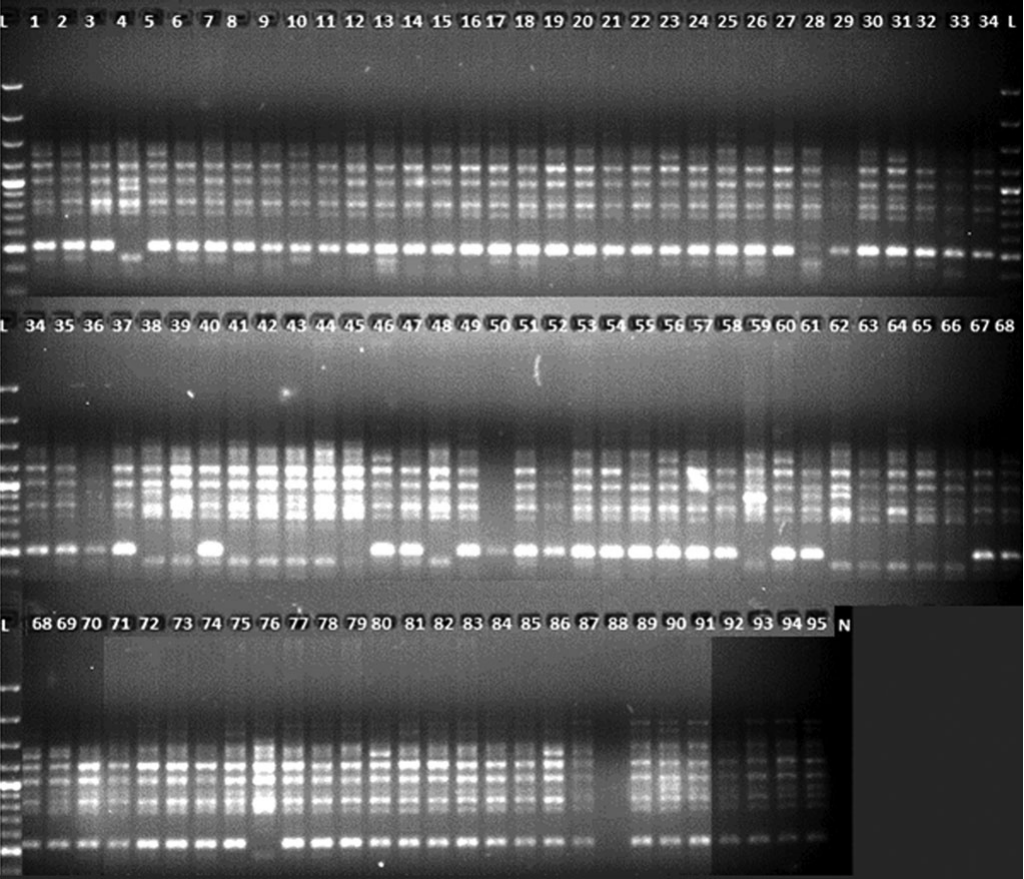
Random amplified polymorphic DNA B5 primer amplification profile of 95 Indian and Turkish wheat genotypes.

Ninety-five Indian and Turkish wheat varieties were amplified using 43 and 27 ISSR and RAPD markers, respectively. The 10 most polymorphic ISSR and RAPD primers generated 116 and 82 genetic loci, respectively, with a total of 198 loci. Among ISSR primers, ISSR M3 generated the maximum number of polymorphic fragments (16) and cRAPD2 was the most prolific RAPD primer (11). In total, 94.8 and 81.7 % bands were found to be polymorphic among ISSR and RAPD markers. The average number of polymorphic bands per primer was 11.0 and 6.7 for ISSR and RAPD primers, respectively (Tables 2 and 3). For both primer types, the main amplified region was in the range of 300–2000 bp (Figs 1 and 2).

### Genetic relationships/association

**Figure 3.**
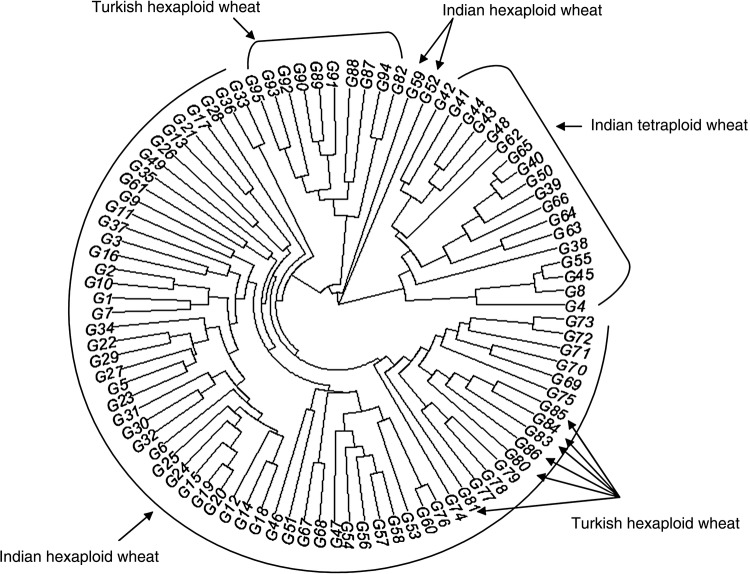
Simple matching coefficient-based Fan dendrogram using NTSYS-PC and R software package of 95 Indian and Turkish wheat genotypes.

A Fan dendrogram of the combined RAPD and ISSR data showed clear groupings of genotypes on the basis of both ploidy and origin (Fig. 3). On combining both RAPD and ISSR data, individual errors of either marker system are reduced and combined the dendrogram provided a more robust overview of the relatedness of Indian and Turkish populations.

On the basis of ploidy, wheat varieties were divided into three clusters, containing 18 tetraploid and 77 hexaploid varieties. Among hexaploid varieties, two Indian genotypes, NW 2036 and RAJ 4027, were separated as outliers from the rest. However, all the hexaploid genotypes were basically divided into two groups, and six Turkish genotypes were clustered with the Indian Hexaploid group. Similarity coefficients among Indian hexaploid varieties ranged from 0.71 to 0.98 while among Turkish hexaploid varieties ranged from 0.42 to 0.95.

Furthermore, the molecular variance factor in both Indian and Turkish populations was compared as a further measure of genetic diversity. Results from AMOVA for geographical origin indicated 77 % genetic variation within populations, while the variation between the populations was 23 % (PhiPT = 0.232; *P* = 0.010). On the basis of ploidy, AMOVA detected higher genetic variation within tetraploid and hexaploid populations (92 %); however, the genetic variation between ploidies was only 8 % (PhiPT = 0.078; *P* = 0.010) (Table 4).

**Table 4.** Analysis of molecular variance in Indian and Turkish wheat populations.

Source of variation	df	Square sum	Variance component	Percentage	Probability
Geographic origin					
Among populations	1	133.52	4.46	23	<0.001
Within populations	93	1370.776	14.74	77	
Ploidy					
Among populations	1	54.21	1.32	8	<0.001
Within populations	93	1450.09	15.59	92	

### Population structure

**Figure 4.**
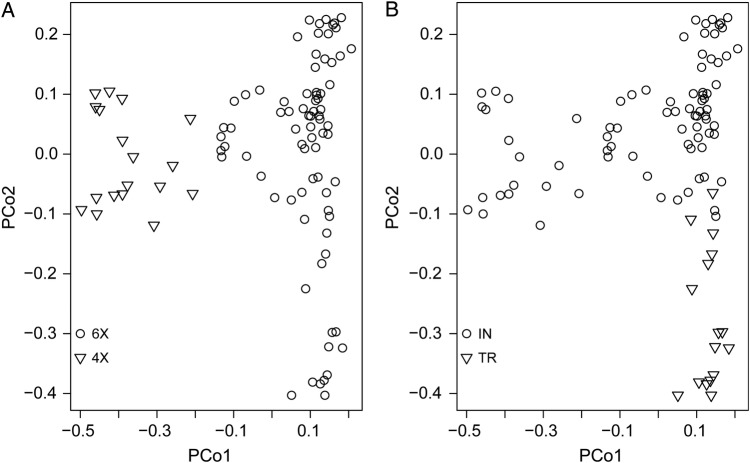
Principal coordinate analysis of 95 Indian and Turkish wheat genotypes based on (A) ploidy level of the genotypes and (B) geographic origin of the genotypes.

Principal coordinate analysis (PCoA) serves as a platform to provide a spatial illustration of the comparative genetic distances between the individuals. It also assesses the robustness of the differentiation among the groups classified by the dendrogram (Liu *et al*. 2013). In our scatterplots, the first two principal components explained 17.6 and 10.7 % of the total variation, respectively. In accordance with the dendrogram, hexaploid individuals were clearly separated from tetraploid varieties by the first principal coordinate (Fig. 4A). Similarly, the second principal coordinate (10.71 % of total variation) divided the Turkish populations from the Indian ones (Fig. 4B).

For population genetic structure analysis, Bayesian clustering modelling was executed in the STRUCTURE software using genotyping data generated by 177 RAPD and ISSR loci. As the clustering model presumes the underlying existence of *K* clusters, an Evano test was performed and yielded *K* = 3 as the highest log-likelihood. This means that 3 was the optimum number of subpopulations, indicating that the two major population groups actually represent three distinct clusters.

**Figure 5.**
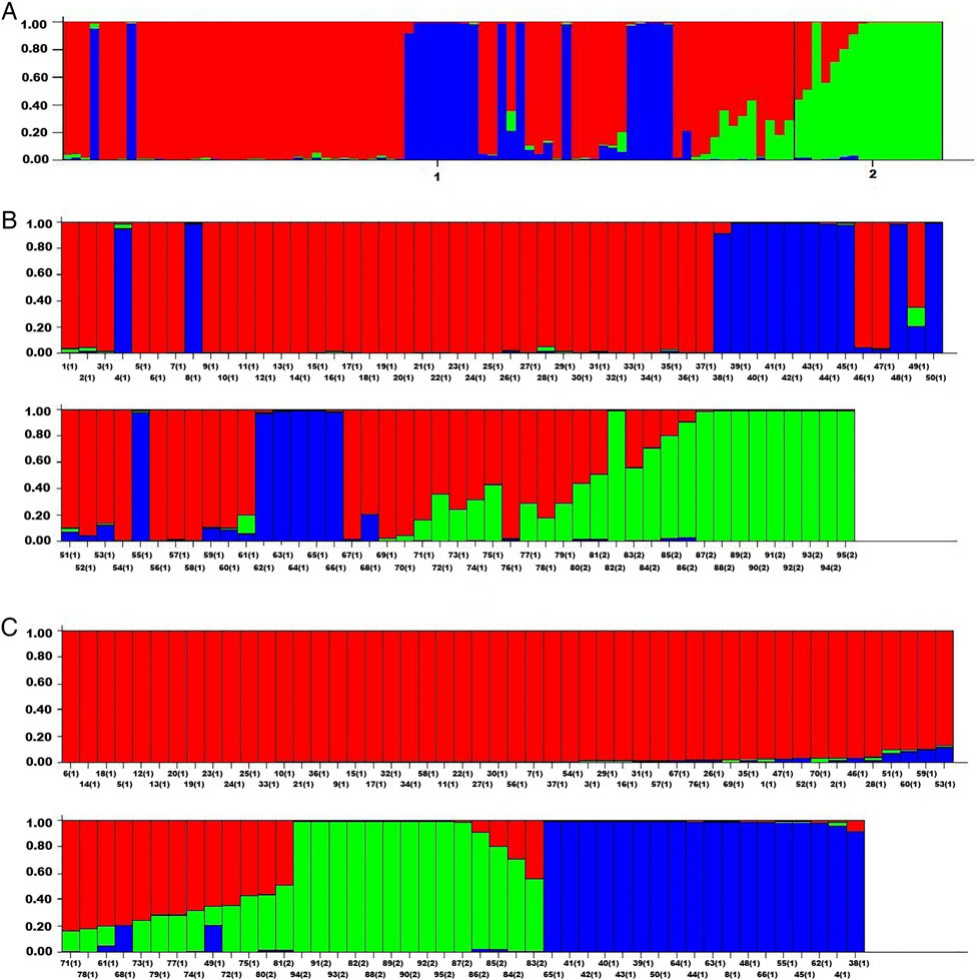
(A) Three clusters inferred from population STRUCTURE analysis; red zone consists of basically Indian varieties with blue zone representing Indian tetraploid subpopulation and green zone includes basically Turkish varieties. (B) For distinctive clusters, vertical coordinates denote membership coefficients and each vertical line along with the horizontal coordinate denotes individual genotypes. Numbers in brackets denote their main population group, India and Turkey. (C) Collection of genotypes on the basis of Q, which explains the proportion of every individual genome that belongs to two distinct clusters.

The analysis of structure according to the geographical origin was performed by setting the range of possible number of subpopulations (*K*) from 1 to 4. Indian and Turkish populations involved in this procedure showed separation from each other in accordance with clusters obtained in PCoA. At *K* = 3, wheat genotypes were divided into three clusters with two main populations, 1 and 2 (Fig. 5A) representing Indian and Turkish wheat gene pool, respectively. Red colour bars represent individuals belonging to the Indian wheat gene pool while those in green belong to the Turkish gene pool. The Indian wheat gene pool was again distributed into subclusters with blue representing the tetraploid wheat population (Fig. 5B). The Indian population consisted of 79 accessions, of which 72 % belonged to the first cluster, 4 % to the second cluster and 24 % to the third; whereas the Turkish group consisted of 16 accessions with 13, 86 and 1 % belonging to the first, second and third cluster, respectively (Table 5). Some of the Indian and Turkish hexaploid genotypes, including NW_2036, RAJ_4027, Bayraktar_2000, Seval, Gün_91, Konya_2002, showed admixture clustering (Fig. 5C). Within the first, second and third clusters, expected heterozygosity within individuals was found to be 0.18, 0.15 and 0.16, respectively.

**Table 5.** Proportion of membership of each pre-defined population in each of the three clusters obtained from STRUCTURE analysis.

Given population	Inferred clusters			Number of individuals
	1	2	3	
1	0.720	0.039	0.241	79
2	0.133	0.857	0.009	16

## Discussion

The complex nature and huge size of the wheat genome pose serious challenges towards genetic means of increasing its production. Hence, furthering our understanding of the wheat genome utilizing a variety of analyses has assisted efforts towards the genetic improvement of modern cultivars. Our examination of the literature to date found no prior genotypic characterization of the Indian and Turkish wheat varieties, simultaneously using RAPD and ISSR markers (Khan *et al*. 2014). The present study constitutes the first attempt to better understand jointly the origin, evolution and molecular diversity of Indian and Turkish wheat varieties at different ploidy levels.

### Evaluating genetic diversity in Indian and Turkish wheat

Since their introduction, ISSR and RAPD markers have been broadly utilized for variability estimation of wheat genotypes. Several RAPD- and ISSR-based diversity studies including diploid, tetraploid and hexaploid wheat have been published (Castagna *et al*. 1997; Nagaoka and Ogihara 1997; Pujar *et al*. 1999, 2002; Barcaccia *et al*. 2002; Teshale *et al*. 2003; Mantzavinou *et al*. 2005; Thomas *et al*. 2006; Aliyev *et al*. 2007; Grewal *et al*. 2007; Anand *et al*. 2008; Cenkci *et al*. 2008; Sawalha *et al*. 2008; Tahir 2008; Carvalho *et al*. 2009; Pandey *et al*. 2012). Due to the possession of diverse (A, B, D) genomes of wheat, tetraploid and hexaploid varieties were involved in the study.

Although Indian and Turkish wheat germplasms were not simultaneously used earlier, the average RAPD- and ISSR primer-based polymorphism, 81.7 and 94.8 %, respectively, revealed in this study, were comparable with several prior diversity studies. The very first attempt made by other researchers among Indian tetraploid wheat varieties revealed high genetic variability in durum released cultivars (50.6 %) in comparison to landraces (44.8 %) (Pujar *et al*. 1999). Teshale *et al*. (2003) found 79.6 % polymorphism among 27 tetra- and hexaploid Indian genotypes using RAPD markers. A detailed study on 96 commercial Indian wheat genotypes, including tetraploids, triticale and hexaploids, indicating 78.8 % polymorphism, revealed a narrow genetic base of tetraploid cultivars in comparison to hexaploids (Thomas *et al*. 2006).

The similarity coefficient values range among Indian hexaploid varieties observed in our study (0.71–0.98) was found to be higher than that in a previous study by Grewal *et al*. (2007) (0.52–0.82) using RAPD markers. In the present work, the average count of polymorphic bands per primer was higher in the case of ISSR (11) compared with that in RAPD (6.7). These results were consistent with a previous study by Pujar *et al*. (2002) on Indian tetraploid wheat varieties. Although limited studies have been performed on diversity assessment of Turkish wheat, Akar and Ozgen (2007) assessed the genetic variability of 100 durum wheat varieties using RAPD markers and observed higher genetic diversity in landraces than in cultivars. Cifci and Yagdi (2012) distinguished 16 Turkish bread wheat varieties using RAPD markers with product size in the range of 300–2800 bp, which was similar to our results.

### Analysis of genetic relationships among wheat genotypes

The dendrogram obtained in this study clearly clustered the genotypes according to their ploidy level, consistently with the evolution of wheat (Alamerew *et al*. 2004). Furthermore, Indian and Turkish varieties were grouped separately. The information revealed by the dendrogram highlighted the parentage association of the varieties. Varieties HD_2177 and HD_2329 grouped together in the dendrogram with 95 % similarity share three common parents, HD_1962, E_4870, K_65. HD_2402 also grouped with its parent variety HD_2236 and showed 96 % similarity. Varieties Raj_1482, Raj_3072 and Raj_3077 were clustered together. Within this cluster, Raj_1482 is the parent of Raj_3077, with 93 % similarity. HD_2307 and HD_2501, which were grouped separately from other ‘HD’ varieties, share as a common parent HD_2160 and consistently exhibited 93 % similarity. Not only hexaploids, but also some of the tetraploid varieties like AKDW_2997 were also allocated in the same subcluster with its parent Raj_1555 and showed 88 % similarity (Fig. 3).

Analysis of molecular variance results disclosed in the study were in agreement with the UPGMA clustering and supported a high level of diversity within-country samples. Although the variation between Indian and Turkish populations was lower in comparison to within-population variation, it was significant according to the partitioning value (*P* = 0.010) (Table 4). The results suggest that similarity association between the countries was affected by within-country inconsistencies of the varieties. This high variation within groups can be attributed to selective adaptation towards the growth conditions at the time of breeding.

### Investigating the Indian and Turkish wheat population structure

Similar separation of Indian and Turkish wheat varieties was observed by PCoA on the basis of ploidy and geographical region. The outcomes of the two methods (cluster analysis and PCoA) were comparable. Both of them classified 95 wheat genotypes mainly into three clusters and offered similar alignment of the genotypes with a few negligible discrepancies. The groups attained were in agreement with the recognized geographical origin as well.

Population structure analyses indicated that wheat accessions can be efficiently categorized on the basis of both geographical origin and ploidy. Using the maximum membership probability in STRUCTURE, Indian and Turkish populations showed similar grouping to the UPGMA and PCoA clustering. The PCoA clustering divided Indian and Turkish populations basically into similar clusters to those produced by the Structure bar plot at *K* = 3. In PCoA, some of the Indian and Turkish varieties showed close association with each other, and similar varieties demonstrated admixture in Structure analysis confirming their relatedness within the diverse gene pool. In dendrogram also, these varieties showed distinct clustering with the main population groups. Closeness of some of the Turkish hexaploid genotypes with Indian hexaploid genotypes in PCoA was also supported by the population structure as well as the dendrogram. Similar and mutually supportive results from all the statistical analyses demonstrated the capability of RAPD and ISSR markers to distinguish Indian and Turkish wheat varieties efficiently.

## Conclusions

Genetic diversity evaluation serves as a crucial platform in plant improvement. The present study provides a detailed understanding of the genetic association of Indian and Turkish hexaploid and tetraploid wheat. The Turkish hexaploid populations showed their closeness to Indian genotypes, confirming their alliance within the diverse gene pool. The present genetic diversity study of wheat material obtained from diverse regions will support breeders in expanding the genetic variation of breeding accessions and utilizing the studied wheat resources more effectively.

## Sources of Funding

M.K.K. has been granted ‘2216 Research Fellowship for Foreign Citizens’ by TÜBİTAK (The Scientific and Technological Research Council of Turkey) for performing the present research work. Also, a part of the research was funded by BAP (Turkish Scientific Research Project agency) under Project No. 14401106.

## Contributions by the Authors

M.K.K. initiated and obtained the funding for the research work. M.K.K. and A.P. contributed in performing the research work and preparation of the manuscript. M.K.K., A.P., S.A.K. and Y.O. carried out all statistical analyses. All other authors have provided suggestions and guidance for the successful completion of the work. All authors read and approved the final manuscript.

## Conflict of Interest Statement

None declared.
